# Drug-Related Problems of Children With Chronic Diseases in a Chinese Primary Health Care Institution: A Cross-Sectional Study

**DOI:** 10.3389/fphar.2022.874948

**Published:** 2022-07-18

**Authors:** Xiao-Feng Ni, Chun-Song Yang, Li-Nan Zeng, Hai-Long Li, Sha Diao, De-Yuan Li, Jin Wu, Yuan-Chun Liu, Zhi-Jun Jia, Guo Cheng, Ling-Li Zhang

**Affiliations:** ^1^ Department of Pharmacy, West China Second University Hospital, Sichuan University, Chengdu, China; ^2^ Evidence-Based Pharmacy Center, West China Second University Hospital, Sichuan University, Chengdu, China; ^3^ Key Laboratory of Birth Defects and Related Diseases of Women and Children, Ministry of Education (Sichuan University), Chengdu, China; ^4^ West China School of Medicine, Sichuan University, Chengdu, China; ^5^ Pediatric Intensive Care, West China Second University Hospital, Sichuan University, Chengdu, China; ^6^ Department of Pediatrics, West China Second University Hospital, Sichuan University, Chengdu, China; ^7^ Zigong Da’an Maternity and, Child Health Care Hospital, Zigong, China; ^8^ West China School of Pharmacy, Sichuan University, Chengdu, China; ^9^ Laboratory of Molecular Translational Medicine, Center for Translational Medicine, Sichuan University, Chengdu, China; ^10^ China Center for Evidence-based Medicine, West China Hospital, Sichuan University, Chengdu, China

**Keywords:** drug-related problems, primary heath care, children, chronic disease, cross-sectional study, China

## Abstract

**Introduction:** Drug-related problems (DRPs) refer to events or circumstances involving drug therapy that actually or potentially interfere with desired health outcomes. DRPs might be severe for children with chronic diseases managed at primary health care institutions, but the relevant research is scarce.

**Objective:** In this cross-sectional study, we aimed to explore the prevalence, types, causes, and influencing factors of DRPs in children with chronic diseases in a Chinese primary health care institution.

**Methods:** We recruited children with chronic diseases who visited the pediatric outpatient department in a primary health care institution from July 1 to 12 October 2021. Clinical pharmacists identified DRPs through medication therapy reviews, classified the types and causes of DRPs, and distinguished the manifested DRPs that affected the outcome and potential DRPs that were going to affect the outcome.

**Results:** A total of 188 children with chronic diseases was included, and 584 DRPs were identified in 89.89% of participants. The most common type of DRPs was “treatment effectiveness” (a manifested problem or potential problem with the effect of the pharmacotherapy; 83.56%), of which 67.29% were potential DRPs. The second common type was “treatment safety” (patient suffers or could suffer from an adverse drug event; 14.21%), of which 89.16% were potential DRPs. The most common cause of DRPs was related to the process of use (42.24%), such as “patient uses/takes less drug than prescribed or does not take the drug at all,” “patient stores drug inappropriately,” and “patient administers/uses the drug in a wrong way.” The second common cause was related to the process of dispensing (29.83%), such as “necessary information not provided or incorrect advice provided” and “prescribed drug is not available.” The third common cause was related to the process of prescribing (26.21%), such as “drug dose is too low” and “no or incomplete drug treatment despite an existing indication.” The number of combined medications was an influencing factor for the frequency of DRPs (*p* < 0.05).

**Conclusion**: This cross-sectional study showed that the current situation regarding DRPs among children with chronic diseases managed in the primary health care institution was serious. The types of DRPs were mainly related to treatment effectiveness, and improper usage of medications was one of the main causes of DRPs. The number of combined drugs was the influencing factor for the frequency of DRPs. In the future, pharmacists should consider formulating pharmaceutical intervention strategies for this specific group according to the characteristics of DRPs.

## 1 Introduction

The Pharmaceutical Care Network Europe Association (PCNE) defined “drug-related problems” (DRPs) as events or circumstances involving drug therapy that actually or potentially interfere with desired health outcomes (Pharmaceutical Care Network Europe Association 2020). DRPs not only focus on the effectiveness and safety of drug therapy but also on the rationality and cost of drug therapy, involving the whole process of prescribing, dispensing, and medication use (Leape et al., 1991; Edwards and Aronson 2000; Morimoto et al., 2004).

As a special population, children may have a higher prevalence of DRPs than adults. A cohort study has shown that the prevalence of potentially harmful medication errors in children was 3 times higher than that in adults (Kaushal et al., 2008). A systematic review has also shown that the prevalence of medication errors was higher in children (53–67%) than in adults (12–59%) (Mira et al., 2015). Studies from Lebanon, Israel, and Australia have shown that pediatric hospital admissions due to DRPs represented 7.9–17.7% (Yosselson-Superstine and Weiss 1982; Easton et al., 1998; Major et al., 1998). Studies from Australia have shown that 4.3% of all hospitalizations were associated with DRPs in children, with an associated medical cost of approximately AUD $181,878.2 (Easton-Carter et al., 2003), while DRPs resulted in a 3.3% emergency visit rate for children, with an associated medical cost of approximately AUD $137,088.74 (Easton et al., 2004).

The prevalence of chronic diseases in children has increased since the 1980s and might further increase (van der Lee et al., 2007; Van Cleave et al., 2010). Chronic diseases usually have a long duration, are prone to relapse, and their therapeutic regimens are relatively complex, which might increase the risk of DRPs. A study using home visits has found that 22% of children with chronic diseases had medication errors when taking medicine at home, which caused manifested or potential harm to children (Walsh et al., 2011). Several studies have shown that the adherence of children and adolescents was usually less than 50% (Quittner et al., 2008). Also, children with complex chronic diseases were more likely to visit emergency departments due to adverse drug events (odds ratio [OR] = 4.76, 95% confidence interval [CI]: 4.45–5.10) (Feinstein et al., 2014). Therefore, compared to other pediatric patients, the risk of DRPs in children with chronic diseases might be increased.

The function of primary health care institutions (PHCIs) is mainly to provide primary public health services, including management and rehabilitation services for common chronic diseases (National Health Commission Of China 2020). In 1978, the international Conference on Primary Health Care set out the goal of health for all (International Conference on Primary Health Care 1978). However, existing studies have shown that PHCIs have some imperfections, such as poor medical quality, underdeveloped pharmaceutical care, and patients’ absent awareness of diseases and medication (Sylvia et al., 2015; Su et al., 2017; Adisa and Omitogun 2019; Khalil and Huang 2020; Li et al., 2020). A systematic review has shown that the median (interquartile range, IQR) of DRPs prevalence among patients in PHCIs was 70.04% (59%), and the median (IQR) of DRPs frequency per patient was 3.4 (2.8) (Ni et al., 2021). Hence, compared to other children with chronic disease, the risk of DRPs among children with chronic disease in PHCIs might be further increased. However, per our systematic retrieval, in primary health care institutions, there are only two studies on the occurrence of DRPs in children without chronic diseases.

Therefore, this cross-sectional study aimed to investigate the characteristics of DRPs among children with chronic disease in a PHCI, including the prevalence, types, causes, and influencing factors of DRPs. The results of this study might provide theoretical evidence to formulate pharmaceutical intervention strategies in the future to ensure the effectiveness and medications safety for children with chronic diseases in PHCIs.

## 2 Methods

### 2.1 Study Design

We conducted a prospective cross-sectional study.

### 2.2 Study Setting

This study was conducted at the Xihanggang Community Health Service Center, which is located in Xihanggang Street, Shuangliu, Chengdu, China. The Xihanggang community covers an area of 38 square kilometers, with 16 communities and 146,000 permanent residents under its jurisdiction (West China Second University Hospital and Sichuan University 2021). This study setting helped ensure a sufficient sample size and diversity.

### 2.3 Study Subjects

Study subjects comprised patients visiting the pediatric outpatient clinic of the Xihanggang Community Health Service Center from July 1 to 12 October 2021. Inclusion criteria were as follows: 1) patients younger than 18 years; 2) patients with at least one diagnosed chronic disease; and 3) patients who were prescribed at least 1 medicine for chronic diseases. Chronic diseases in children are defined in these terms (Mokkink et al., 2008): 1) the disease is not (yet) curable or is highly resistant to treatment, and 2) the disease has been present for longer than 3 months, it will probably last longer than 3 months, or it has occurred 3 times or more during the past year and will probably reoccur. Exclusion criteria were as follows: 1) patients with incomplete clinical data without which statistical analysis cannot be performed; and 2) a refusal to sign the informed consent by patients or their guardian.

### 2.4 Identification of Drug-related problems

Through the children’s health management project of West China Women and Children’s Alliance, the Xihanggang Community Health Service Center cooperated with the West China Second Hospital of Sichuan University (a grade III class A specialized hospital for women and children). Clinical pharmacists from the West China Second Hospital of Sichuan University led the graduate students majoring in clinical pharmacy to identify, classify, and double-check DRPs, but they did not intervene in physicians, pharmacists, and patients’ behaviors in this PHCI. The identification of DRPs mainly was based on package inserts of drugs, authoritative guidelines, the latest editions of textbooks, and approved and documented off-label usage in the hospital. The researchers also used certain software and websites to identify DRPs, such as UpToDate, MCDEX, and Micromedex.

At the first visit, for children who met the inclusion criteria and signed the informed consent, the researchers collected the basic information about children, caregivers, and doctors (such as the sex, age, quality of life, growth and development of children; sex, age, and education level of caregivers; and the working years, professional title, and education level of doctors), data on children’s diseases and medications, including prescription and over-the-counter drugs, through interviews and medical records. In the prescribing process, the researchers compiled a comprehensive list of medications for each patient to identify DRPs in drug selection, form, dosage, and duration. The researchers identified DRPs of the dispensing process by observing the patients’ visits to their doctors and picking up of their medicines at the pharmacy and conducting follow-ups on the eighth day after their first visit. In the dispensing process, the researchers mainly reviewed whether there were shortages of prescribed drugs, inadequate or incorrect medication information, or wrong drug or strength provision. In the use process, the researchers created a comprehensive list of medication therapy reviews for each patient; thus, possible DRPs could be reviewed item by item during the follow-ups. We formulated a unified follow-up record table in which the questions were designed based on the Pharmaceutical Care Network Europe (PCNE)-DRP classification system v9.1 to standardize the follow-ups (Pharmaceutical Care Network Europe Association 2020).

### 2.5 Outcomes

The following outcomes were investigated: 1) the prevalence of DRPs; 2) the types and causes of DRPs; 3) the influencing factors related to the frequency of DRPs. Regarding the types and causes of DRPs, the primary domain and subdomain types and causes of DRPs were classified according to the PCNE-DRP classification system v. 9.1. Also, the manifested DRPs (DRPs that affected the outcome) or potential DRPs (DRPs that were going to affect the outcome) were classified.

### 2.6 Sample Size

The sample size was calculated with the primary outcome “the prevalence of DRPs.” and the calculation formula was $\text{n}={\left(\frac{Z_{1-\text{a}/2}}{\delta }\right)}^{2}\times p\times \left(1-p\right)$ (Newcombe 1998; Fleiss 2003), where a = 0.05, Z_1-α/2_ = 1.96. *P* is the expected prevalence of DRPs. According to the results of a systematic review (Ni et al., 2021), the prevalence of DRPs in PHCIs has been about 70.04%, and the allowable error *δ* = 0.07; therefore, the calculated n is 163. According to the preliminary investigation experience, the rate of loss to follow-ups was 15%; thus, 192 children should be recruited.

### 2.7 Data Statistics

Excel 2020 was used to organize the data, and SPSS 24.0 was used for statistical analysis. The continuous data were described by mean ± standard deviation (X ± s), and the categorical variables were described by frequency and percent of the total sample (%). Missing values were filled in based on central trends among the samples; for example, numerical variables were filled with the mean or median, and fractional variables were filled with the mode (Austin et al., 2021). Poisson regression analysis of potential factors that may affect the frequency of DRPs was performed. The outcome measure was the number of DRPs per patient. Patient-related predictors included gender, age, growth and development, quality of life, education level, residence, type of visits, and number of combined medications. Primary caregiver-related predictors included gender, age, education level, relationship with children, knowledge level of chronic diseases, knowledge level of medications, payment method of medical expenses, and family per capita monthly income.

### 2.8 Ethical Approval

This study obtained the approval of the Medical Ethics Committee of the West China Second University Hospital of Sichuan University before the formal implementation (identifier: MR 2021 ERA No.136).

### 2.9 Study Registration

This study was registered on the website of the Chinese Clinical Trial Registry (identifier: ChiCTR2100048235).

## 3 Results

During the study period, a total of 234 children met the inclusion criteria, while 46 children among them met the exclusion criteria (26 children or their primary caregivers refused to sign informed consent forms, 13 children’s medical records were missing critical information, 6 children were lost to follow-up, and 1 children visited the doctor repeatedly during the observation period). Finally, 188 children were included in the analysis.

### 3.1 Basic Characteristics

Of all participants, 52.66% (*n* = 99) were males, 121 children (64.36%) were aged 3–6 years, and 100 children (53.19%) were in kindergarten (*n* = 100, 53.19%). Most of participants had normal growth and development (*n* = 138, 73.40%), and quality of life score of ≥95 (*n* = 167, 88.83%). A total of 49.47% of children visited the doctor for the first time due to chronic disease. The participants’ chronic diseases were mostly respiratory (*n* = 170, 90.43%), and most of them were complicated with two or more diseases (*n* = 119, 63.30%) and took two or more drugs simultaneously (*n* = 163, 86.70%). Furthermore, most participants lived in urban areas (*n* = 176, 93.62%), and most of the medical expenses were paid at their own expense (*n* = 183, 97.34%).

Most of the primary caregivers were female (*n* = 163, 86.70%), and most of them were parents of children (*n* = 159, 84.57%), aging 30–40 years (*n* = 108, 57.45%). Half of the primary caregivers had junior college or bachelor’s degrees (*n* = 94, 50.00%), and half of them had a family per capita monthly income of more than 5,000 yuan (*n* = 93, 50.53%). Their knowledge levels of chronic diseases (*n* = 83, 44.15%) and knowledge levels of medications (*n* = 62, 32.98%) were mainly “general”.

The working years of the receiving doctors were mostly 30–40 years (*n* = 73, 38.83%), and their professional titles were mainly chief physicians (*n* = 89, 47.34%). All doctors had bachelor’s degrees (*n* = 188, 100%).

The detailed basic information on the participants, primary caregivers, and receiving doctors is shown in Table 1.

**TABLE 1:** Basic information of the participants, primary caregivers, and receiving doctors.

Basic Information	n	Percentage (%)	Basic Information	n	Percentage (%)
Gender of participants			Gender of primary caregivers		
Male	99	52.66	Male	163	86.70
Female	89	47.34	Female	25	13.30
Age of participants (years)			Age of primary caregivers (years)		
Infant (28 days–2 years)	48	25.53	<30	41	21.81
Children (3–6)	121	64.36	30–40	108	57.45
Older children (7–12)	19	10.11	40–50	12	6.38
Growth and development of participants			≥50	27	14.36
Below normal	15	7.98	Education level of primary caregivers		
Normal	138	73.40	Junior high or below	47	25.00
Above normal	12	6.38	High school or technical secondary school	43	22.87
NR	23	12.23	Junior college or bachelor	94	50.00
Quality of life score of participants			Postgraduate or above	4	2.31
≥95	167	88.83	Primary caregivers’ relationship with participants		
<95	21	11.17	Parents	159	84.57
Education level of participants			Grandparents	29	15.43
Preschool	48	25.53	Primary caregivers’ knowledge level of chronic diseases		
Kindergarten	100	53.19	Very poor	10	5.32
Primary school	40	21.28	Poor	60	31.91
Residence			General	83	44.15
Urban areas	176	93.62	Well	29	15.43
Rural areas	12	6.38	Very well	6	3.19
Type of visits			Primary caregivers’ knowledge level of medications		
First visit	93	49.47	Very poor	23	12.23
Subsequent visit	95	50.53	Poor	59	31.38
Type of chronic diseases			General	62	32.98
Respiratory system	170	90.43	Well	34	18.09
Skin and subcutaneous tissue	14	7.45	Very well	10	5.32
Digestive system	7	3.72	Payment method of medical expenses		
Blood system	1	0.53	Self-paying	183	97.34
Number of complications			Medical insurance	5	2.66
1	69	36.70	Family per capita monthly income		
2	93	49.47	<3000	18	9.57
3	25	13.30	3000–5,000	75	39.89
4	1	0.53	≥5,000	93	50.53
Number of combined medicines			NR	2	1.06
1–2	70	37.23	Working years of receiving doctors (years)		
3–4	102	54.26	<10	51	27.13
5–6	16	8.51	10–20	48	25.53
Professional title of receiving doctors			20–30	16	8.51
Attending physician	77	40.96	30–40	73	38.83
Associate chief physician	22	11.70	Education level of receiving doctors		
Chief physician	89	47.34	Undergraduate	188	100.00

Note: NR, not report.

### 3.2 Occurrence of Drug-Related Problems

**FIGURE 1:**
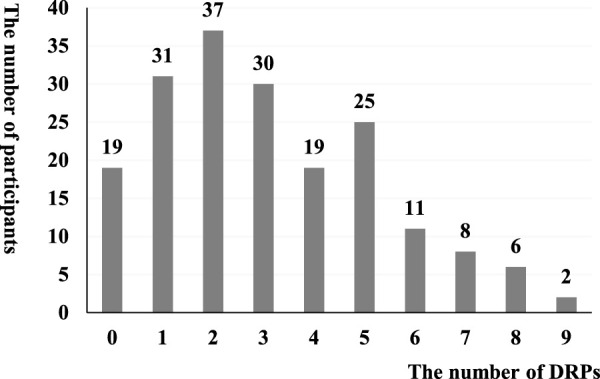
The number distribution of DRPs in children with chronic diseases in the PHCI.

Among 188 children included in the analysis, there were 584 DRPs, with an average of 3.11 ± 2.19 DRPs. A total of 169 children had at least 1 DRP, with a DRP prevalence of 89.89%. The frequency distribution of DRPs is shown in Figure 1.

### 3.3 Types and Causes of Drug-Related Problems

The most common type of DRPs represented the primary domain “P1: treatment effectiveness” (488 DRPs, 83.56%). The common primary subdomains were “P1.2: effect of drug treatment not optimal” (447 DRPs, 76.54%) and “P1.1: no effect of drug treatment despite correct use” (39 DRPs, 6.68%), while out of these, potential DRPs accounted for 80.53%. The second common type of DRPs was “P2: treatment safety” (83 DRPs, 14.21%), including “P2.1 adverse drug events occurred or may occur,” and most of them were potential DRPs (74 DRPs, 89.16%). Table 2 shows the type distribution of DRPs.

**TABLE 2:** The type distribution of DRPs in children with chronic diseases in the PHCI.

Type	Manifest DRPs (n, %)	Potential DRPs (n, %)	Total (n, %)
P1 Treatment effectiveness	95 (16.27)	393 (67.29)	488 (83.56)
P1.1 No effect of drug treatment despite the correct use	38 (6.51)	1 (0.17)	39 (6.68)
P1.2 Effect of drug treatment not optimal	55 (9.42)	392 (67.12)	447 (76.54)
P1.3 Untreated symptoms or indication	2 (0.34)	0 (0.00)	2 (0.34)
P2 Treatment safety	9 (1.54)	74 (12.67)	83 (14.21)
P2.1 Adverse drug event (possibly) occurring	9 (1.54)	74 (12.67)	83 (14.21)
P3 Other	13 (2.23)	0 (0.00)	13 (2.23)
P3.1 Unnecessary drug-treatment	13 (2.23)	0 (0.00)	13 (2.23)

According to the classification of different processes, DRPs mainly represented the process of use (245 DRPs, 42.24%), followed by the process of dispensing (173 DRPs, 29.83%), the process of prescription and drug selection (152 DRPs, 26.21%), and other processes (10 DRPs, 1.72%). In the process of prescription, the common causes included “C3.1 drug dose too low” (61 DRPs, 10.45%), “C1.5 no or incomplete drug treatment despite existing indication” (38 DRPs, 6.51%), and “C3.3 dosage regimen is not frequent enough” (21 DRPs, 3.60%). In the process of dispensing, the common causes included “C5.2 necessary information not provided or incorrect advice provided” (114 DRPs, 19.52%) and “C5.1 prescribed drug not available” (62 DRPs, 10.62%). In the process of use, the common causes included “C7.1 patient intentionally uses/takes less drug than prescribed or does not take the drug at all for whatever reason” (82 DRPs, 14.04%), “C7.6 patient stores drug inappropriately” (81 DRPs, 13.87%), and “C7.8 patient unintentionally administers/uses the drug in a wrong way” (69 DRPs, 11.82%). Among other processes, the main cause was suspected adverse drug reactions (9 DRPs, 1.54%). Table 3 demonstrates the causes distribution of DRPs.

**TABLE 3:** The causes distribution of DRPs in children with chronic diseases in the PHCI.

Process	Cause	Total (n, %)
Prescribing and drug selection	C1 Drug selection	53 (9.08%)
	C1.2 No indication for a drug	13 (2.23%)
	C1.4 Inappropriate duplication of a therapeutic group or active ingredient	2 (0.34%)
	C1.5 No or incomplete drug treatment in spite of existing indication	38 (6.51%)
	C2 Drug form	8 (1.37%)
	C2.1 Inappropriate drug form/formulation (for this patient)	8 (1.37%)
	C3 Dose selection	90 (15.41%)
	C3.1 Drug dose too low	61 (10.45%)
	C3.2 Drug dose of a single active ingredient too high	8 (1.37%)
	C3.3 Dosage regimen is not frequent enough	21 (3.60%)
	C4 Treatment duration	2 (0.34%)
	C4.2 Duration of treatment too long	2 (0.34%)
Disp	C5 Dispensing	176 (30.14%)
	C5.1 Prescribed drug not available	62 (10.62%)
	C5.2 Necessary information not provided or incorrect advice provided	114 (19.52%)
Use	C7 Patient related	245 (41.95%)
	C7.1 Patient intentionally uses/takes less drug than prescribed or does not take the drug at all for whatever reason	82 (14.04%)
	C7.2 Patient uses/takes more drug than prescribed	3 (0.51%)
	C7.4 Patient decides to use an unnecessary drug	3 (0.51%)
	C7.5 Patient takes food that interacts	3 (0.51%)
	C7.6 Patient stores drug inappropriately	81 (13.87%)
	C7.7 Inappropriate timing or dosing intervals	3 (0.51%)
	C7.8 Patient unintentionally administers/uses the drug in a wrong way	69 (11.82%)
	C7.9 Patient physically unable to use drug/form as directed	1 (0.17%)
Other	C9 Other	10 (1.7%)
	C9.2 Suspected adverse drug reactions	9 (1.54%)
	C9.3 No obvious cause	1 (0.17%)

### 3.4 Poisson Regression of Potential Factors Associated With the Frequency of Drug-Related Problems

Poisson regression of factors that may influence the frequency of detected DRPs is shown in Table 4. The goodness-of-fit Chi squared test showed that the regression model fits reasonably well (deviance divided by degrees of freedom of 1.258). There was a significant 36.9% increase in the frequency of DRPs detected for each additional medicine added to a patient’s treatment regimen.

**TABLE 4:** Poisson regression of potential factors associated with thefrequency of detected DRPs.

Variables	β	Standard Error	EXP(β)	95% *CI*		*P*
				Lower Limit	Upper Limit	
Variables associated with children						
Gender	0.057	0.09	1.059	-0.119	0.234	0.523
Age	0.136	0.1082	1.146	-0.076	0.348	0.209
Growth and development	-0.080	0.1178	0.924	-0.310	0.151	0.499
Quality of life score	0.155	0.1406	1.167	-0.121	0.430	0.271
Residence	0.003	0.2006	1.003	-0.390	0.396	0.988
Education level	-0.170	0.0949	0.844	-0.356	0.017	0.074
Type of visit	-0.040	0.0906	0.961	-0.217	0.138	0.660
Number of combined medications	0.314	0.0419	1.369	0.232	0.396	0.000
Variables associated with primary caregivers						
Gender	0.100	0.1310	1.105	-0.157	0.357	0.446
Age	0.077	0.0966	1.081	-0.112	0.267	0.423
Relationship with children	0.072	0.2627	1.074	-0.443	0.586	0.785
Education level	-0.078	0.0663	0.925	-0.208	0.052	0.240
Family per capita monthly income	0.112	0.0769	1.118	-0.039	0.263	0.145
Payment method of medical expenses	-0.258	0.2384	0.773	-0.725	0.210	0.280
Knowledge level of chronic diseases	-0.059	0.0632	0.943	-0.183	0.065	0.351
Knowledge level of medications	0.007	00534	1.007	-0.099	0.113	0.897
Variables associated with children						
Gender	0.057	0.09	1.059	-0.119	0.234	0.523
Age	0.136	0.1082	1.146	-0.076	0.348	0.209
Growth and development	-0.080	0.1178	0.924	-0.310	0.151	0.499
Quality of life score	0.155	0.1406	1.167	-0.121	0.430	0.271
Residence	0.003	0.2006	1.003	-0.390	0.396	0.988
Education level	-0.170	0.0949	0.844	-0.356	0.017	0.074
Type of visit	-0.040	0.0906	0.961	-0.217	0.138	0.660
Number of combined medications	0.314	0.0419	1.369	0.232	0.396	0.000
Variables associated with primary caregivers						
Gender	0.100	0.1310	1.105	-0.157	0.357	0.446
Age	0.077	0.0966	1.081	-0.112	0.267	0.423
Relationship with children	0.072	0.2627	1.074	-0.443	0.586	0.785
Education level	-0.078	0.0663	0.925	-0.208	0.052	0.240
Family per capita monthly income	0.112	0.0769	1.118	-0.039	0.263	0.145
Payment method of medical expenses	-0.258	0.2384	0.773	-0.725	0.210	0.280
Knowledge level of chronic diseases	-0.059	0.0632	0.943	-0.183	0.065	0.351
Knowledge level of medications	0.007	00534	1.007	-0.099	0.113	0.897

## 4 Discussion

### 4.1 Findings of this Study

In this study, we investigated the occurrence, types, causes, and influencing factors of DRPs in children with chronic diseases in a PHCI. The results showed that the prevalence of DRPs in children with chronic disease in the PHCI was 89.89%, with an average frequency of 3.11 ± 2.19 DRPs. Compared with the results of the systematic review of patients in PHCIs mentioned earlier (Ni et al., 2021), children with chronic disease, as a special population in PHCIs, might have a more difficult situation regarding DRPs, requiring more attention from pharmacists. Similarly, the prevalence of DRPs in children in non-PHCIs has ranged from 21 to 51.2% (Birarra et al., 2017; Rashed et al., 2013; Rashed et al., 2012; Easton-Carter et al., 2003), which might be not as bad as that in PHCIs. In this study, treatment effectiveness was the most common type of DRPs, mainly characterized by a poor treatment effect. Treatment safety was the second most common type of DRPs, including manifested or potential adverse drug events. The process of use was the main cause of DRPs in this study. In pediatric patients, their use of medications is greatly influenced by their primary caregivers (Solanki et al., 2017; Wang et al., 2020). From the basic characteristics of primary caregivers in this study, nearly half of primary caregivers’ education levels was high school or below, with poor knowledge of the chronic diseases and medications, which might have aggravated the occurrence of DRPs. The process of dispensing was also one of the important causes of DRPs, mainly containing inadequate medication information provided by pharmacists and drug shortages. Considering these causes, continuing education for pharmacists in PHCIs will be important to reduce the occurrence of DRPs in the future. The problem of drug shortages in PHCIs is very common (Bawontuo et al., 2021). Drug shortage not only restricts patients from choosing the most appropriate treatment regimes and affects their treatment effect but also induces patients to purchase medications from private pharmacies by themselves, which might provide hidden risks to medication safety. Therefore, it is urgent to improve the accessibility of clinically essential medications in PHCIs. Although the diseases of patients in PHCIs are usually relatively common and simple, the process of prescription was still one of the common causes of DRPs in our research, probably because of the limited qualification level of receiving doctors. It is necessary to create a medical alliance or cooperation; therefore, doctors in PHCIs will be led by clinically experienced doctors in the upper-level hospitals to improve their diagnosis and treatment quality. This study also explored the influencing factors. Similar to several other studies on pediatric patients (Birarra et al., 2017; Rashed et al., 2012; Easton-Carter et al., 2003), the number of combined medications was an independent factor associated with the occurrence of DRPs, possibly because more complicated therapy regimen leads to more adverse drug reactions, drug-drug interactions, medication errors, and non-adherence.

### 4.2 Innovation of this Research

One of the responsibilities of PHCIs is to undertake the long-term management of patients with common chronic diseases. Through the previous systematic retrieval, we found that research on the DRPs in children with chronic diseases in PHCIs was lacking. Therefore, this was the first study to focus on this special population to explore the occurrence, types, causes, and influence factors of the DRPs, which would help formulate pharmaceutical intervention strategies to reduce the occurrence of DRPs.

### 4.3 Limitations of this Research

Firstly, this study is a single-center cross-sectional survey; thus, the results may lack universality. However, the PHCI selected as the research site is a community hospital designated by 16 communities, and in 2020, the number of pediatric emergency/outpatient visits reached more than 120,000. Therefore, the sample of children is representative to a certain extent. Secondly, since this study is cross-sectional, the investigation of the influencing factors of DRPs is only a preliminary exploration, and the results still need to be verified by a large sample cohort study.

### 4.4 Future Research Direction

Firstly, to solve the defects of this study, large-sample multi-center studies can be conducted in the future to further explore the characteristics of DRPs in children with chronic diseases in PHCIs, which is conducive to the development of the DRPs risk assessment model. Secondly, the method to provide a pharmaceutical intervention aimed at DRPs in children with chronic disease patients in PHCIs will be an important direction for the next step of research to ensure that these children can obtain high-quality chronic disease management and rehabilitation services even if they are in PHCIs.

## 5 Conclusion

This cross-sectional study investigated the DRPs in children with chronic diseases in a PHCI and found that the prevalence of DRPs and the frequency of DRPs per capita were significantly high. The types of DRPs mainly involved treatment effectiveness, and improper usage of medications was one of the main causes of DRPs. The number of combined drugs was the influencing factor for the frequency of DRPs. In the future, it is urgent to formulate DRPs pharmaceutical intervention strategies for children with chronic diseases in PHCIs according to the common types, causes, and influencing factors of DRPs in this population.

## Data Availability

The original contributions presented in the study are included in the article/Supplementary Material, further inquiries can be directed to the corresponding authors.

## Abbreviations

DRP, drug-related problem; IQR, interquartile range; PCNE, Pharmaceutical Care Network Europe; PHCI, primary health care institution.
