# Supplementary material for: Drug-Related Problems of Children With Chronic Diseases in a Chinese Primary Health Care Institution: A Cross-Sectional Study
Source: Front Pharmacol. 2022 Jul 18;13:874948. doi: 10.3389/fphar.2022.874948 (PMC9342849; doi:10.3389/fphar.2022.874948)
Supplement: Supplementary file 1 [file Table1.docx]

**Table 1 Basic information of the participants, primary caregivers, and receiving doctors**

| Basic information | n | Percentage (%) | Basic information | n | Percentage (%) |
| --- | --- | --- | --- | --- | --- |
| Gender of participants |  |  | Gender of primary caregivers | | |
| Male | 99 | 52.66% | Male | 163 | 86.70% |
| Female | 89 | 47.34% | Female | 25 | 13.30% |
| Age of participants (years) | | | Age of primary caregivers (years) | | |
| Infant (28 days–2 years) | 48 | 25.53% | <30 | 41 | 21.81% |
| Children (3–6) | 121 | 64.36% | 30–40 | 108 | 57.45% |
| Older children (7–12) | 19 | 10.11% | 40–50 | 12 | 6.38% |
| Growth and development of participants | | | ≥50 | 27 | 14.36% |
| Below normal | 15 | 7.98% | Education level of primary caregivers | | |
| Normal | 138 | 73.40% | Junior high or below | 47 | 25.00% |
| Above normal | 12 | 6.38% | High school or technical secondary school | 43 | 22.87% |
| NR | 23 | 12.23% | Junior college or bachelor | 94 | 50.00% |
| Quality of life score of participants | | | Postgraduate or above | 4 | 2.31% |
| ≥95 | 167 | 88.83% | Primary caregivers’ relationship with participants | | |
| <95 | 21 | 11.17% | Parents | 159 | 84.57% |
| Education level of participants | | | Grandparents | 29 | 15.43% |
| Preschool | 48 | 25.53% | Primary caregivers’ knowledge level of chronic diseases | | |
| Kindergarten | 100 | 53.19% | Very poor | 10 | 5.32% |
| Primary school | 40 | 21.28% | Poor | 60 | 31.91% |
| Residence |  |  | General | 83 | 44.15% |
| Urban areas | 176 | 93.62% | Well | 29 | 15.43% |
| Rural areas | 12 | 6.38% | Very well | 6 | 3.19% |
| Type of visits |  |  | Primary caregivers’ knowledge level of medications | | |
| First visit | 93 | 49.47% | Very poor | 23 | 12.23% |
| Subsequent visit | 95 | 50.53% | Poor | 59 | 31.38% |
| Type of chronic diseases |  |  | General | 62 | 32.98% |
| Respiratory system | 170 | 90.43% | Well | 34 | 18.09% |
| Skin and subcutaneous tissue | 14 | 7.45% | Very well | 10 | 5.32% |
| Digestive system | 7 | 3.72% | Payment method of medical expenses | | |
| Blood system | 1 | 0.53% | Self-paying | 183 | 97.34% |
| Number of complications |  |  | Medical insurance | 5 | 2.66% |
| 1 | 69 | 36.70% | Family per capita monthly income | | |
| 2 | 93 | 49.47% | < 3000 | 18 | 9.57% |
| 3 | 25 | 13.30% | 3000–5000 | 75 | 39.89% |
| 4 | 1 | 0.53% | ≥ 5000 | 93 | 50.53% |
| Number of combined medicines | | | NR | 2 | 1.06% |
| 1–2 | 70 | 37.23% | Working years of receiving doctors (years) | | |
| 3–4 | 102 | 54.26% | < 10 | 51 | 27.13% |
| 5–6 | 16 | 8.51% | 10–20 | 48 | 25.53% |
| Professional title of receiving doctors | | | 20–30 | 16 | 8.51% |
| Attending physician | 77 | 40.96% | 30–40 | 73 | 38.83% |
| Associate chief physician | 22 | 11.70% | Education level of receiving doctors | | |
| Chief physician | 89 | 47.34% | Undergraduate | 188 | 100.00% |

Note: NR, not report.
