# Supplementary material for: Drug-Related Problems of Children With Chronic Diseases in a Chinese Primary Health Care Institution: A Cross-Sectional Study
Source: Front Pharmacol. 2022 Jul 18;13:874948. doi: 10.3389/fphar.2022.874948 (PMC9342849; doi:10.3389/fphar.2022.874948)
Supplement: Supplementary file 2 [file Table2.docx]

**Table 2 The** **type distribution of DRPs in children with chronic diseases** **in the PHCI**

| Type | Manifest  DRPs (n, %) | Potential  DRPs (n, %) | Total (n, %) |
| --- | --- | --- | --- |
| P1 Treatment effectiveness | 95 (16.27) | 393 (67.29) | 488 (83.56) |
| P1.1 No effect of drug treatment despite the correct use | 38 (6.51) | 1 (0.17) | 39 (6.68) |
| P1.2 Effect of drug treatment not optimal | 55 (9.42) | 392 (67.12) | 447 (76.54) |
| P1.3 Untreated symptoms or indication | 2 (0.34) | 0 (0.00) | 2 (0.34) |
| P2 Treatment safety | 9 (1.54) | 74 (12.67) | 83 (14.21) |
| P2.1 Adverse drug event (possibly) occurring | 9 (1.54) | 74 (12.67) | 83 (14.21) |
| P3 Other | 13 (2.23) | 0 (0.00) | 13 (2.23) |
| P3.1 Unnecessary drug-treatment | 13 (2.23) | 0 (0.00) | 13 (2.23) |
