# Supplementary material for: Drug-Related Problems of Children With Chronic Diseases in a Chinese Primary Health Care Institution: A Cross-Sectional Study
Source: Front Pharmacol. 2022 Jul 18;13:874948. doi: 10.3389/fphar.2022.874948 (PMC9342849; doi:10.3389/fphar.2022.874948)
Supplement: Supplementary file 3 [file Table3.docx]

**Table 3 The causes distribution of DRPs in children with chronic diseases in the PHCI**

| Process | Cause | Total (n, %) |
| --- | --- | --- |
| Prescribing and drug selection | C1 Drug selection | 53 (9.08%) |
|  | C1.2 No indication for a drug | 13 (2.23%) |
|  | C1.4 Inappropriate duplication of a therapeutic group or active ingredient | 2 (0.34%) |
|  | C1.5 No or incomplete drug treatment in spite of existing indication | 38 (6.51%) |
|  | C2 Drug form | 8 (1.37%) |
|  | C2.1 Inappropriate drug form/formulation (for this patient) | 8 (1.37%) |
|  | C3 Dose selection | 90 (15.41%) |
|  | C3.1 Drug dose too low | 61 (10.45%) |
|  | C3.2 Drug dose of a single active ingredient too high | 8 (1.37%) |
|  | C3.3 Dosage regimen is not frequent enough | 21 (3.60%) |
|  | C4 Treatment duration | 2 (0.34%) |
|  | C4.2 Duration of treatment too long | 2 (0.34%) |
| Disp | C5 Dispensing | 176 (30.14%) |
|  | C5.1 Prescribed drug not available | 62 (10.62%) |
|  | C5.2 Necessary information not provided or incorrect advice provided | 114 (19.52%) |
| Use | C7 Patient related | 245 (41.95%) |
|  | C7.1 Patient intentionally uses/takes less drug than prescribed or does not take the drug at all for whatever reason | 82 (14.04%) |
|  | C7.2 Patient uses/takes more drug than prescribed | 3 (0.51%) |
|  | C7.4 Patient decides to use an unnecessary drug | 3 (0.51%) |
|  | C7.5 Patient takes food that interacts | 3 (0.51%) |
|  | C7.6 Patient stores drug inappropriately | 81 (13.87%) |
|  | C7.7 Inappropriate timing or dosing intervals | 3 (0.51%) |
|  | C7.8 Patient unintentionally administers/uses the drug in a wrong way | 69 (11.82%) |
|  | C7.9 Patient physically unable to use drug/form as directed | 1 (0.17%) |
| Other | C9 Other | 10 (1.7%) |
|  | C9.2 Suspected adverse drug reactions | 9 (1.54%) |
|  | C9.3 No obvious cause | 1 (0.17%) |
