# Supplementary material for: Drug-Related Problems of Children With Chronic Diseases in a Chinese Primary Health Care Institution: A Cross-Sectional Study
Source: Front Pharmacol. 2022 Jul 18;13:874948. doi: 10.3389/fphar.2022.874948 (PMC9342849; doi:10.3389/fphar.2022.874948)
Supplement: Supplementary file 4 [file Table4.docx]

**Table 4 Poisson regression of potential factors associated with the**

**frequency of detected DRPs**

| Variables | β | Standard error | EXP(β) | 95% *CI* | |  | *P* |
| --- | --- | --- | --- | --- | --- | --- | --- |
|  |  |  |  | Lower limit | Upper limit |  |  |
| Variables associated with children | | | | | | | |
| Gender | 0.057 | 0.09 | 1.059 | -0.119 | 0.234 |  | 0.523 |
| Age | 0.136 | 0.1082 | 1.146 | -0.076 | 0.348 |  | 0.209 |
| Growth and development | -0.080 | 0.1178 | 0.924 | -0.310 | 0.151 |  | 0.499 |
| Quality of life score | 0.155 | 0.1406 | 1.167 | -0.121 | 0.430 |  | 0.271 |
| Residence | 0.003 | 0.2006 | 1.003 | -0.390 | 0.396 |  | 0.988 |
| Education level | -0.170 | 0.0949 | 0.844 | -0.356 | 0.017 |  | 0.074 |
| Type of visit | -0.040 | 0.0906 | 0.961 | -0.217 | 0.138 |  | 0.660 |
| Number of combined medications | 0.314 | 0.0419 | 1.369 | 0.232 | 0.396 |  | 0.000 |
| Variables associated with primary caregivers | | | | | | | |
| Gender | 0.100 | 0.1310 | 1.105 | -0.157 | 0.357 |  | 0.446 |
| Age | 0.077 | 0.0966 | 1.081 | -0.112 | 0.267 |  | 0.423 |
| Relationship with children | 0.072 | 0.2627 | 1.074 | -0.443 | 0.586 |  | 0.785 |
| Education level | -0.078 | 0.0663 | 0.925 | -0.208 | 0.052 |  | 0.240 |
| Family per capita monthly income | 0.112 | 0.0769 | 1.118 | -0.039 | 0.263 |  | 0.145 |
| Payment method of medical expenses | -0.258 | 0.2384 | 0.773 | -0.725 | 0.210 |  | 0.280 |
| Knowledge level of chronic diseases | -0.059 | 0.0632 | 0.943 | -0.183 | 0.065 |  | 0.351 |
| Knowledge level of medications | 0.007 | 00534 | 1.007 | -0.099 | 0.113 |  | 0.897 |
